# Metabolic models and gene essentiality data reveal essential and conserved metabolism in prokaryotes

**DOI:** 10.1371/journal.pcbi.1006556

**Published:** 2018-11-16

**Authors:** Joana C. Xavier, Kiran Raosaheb Patil, Isabel Rocha

**Affiliations:** 1 Department of Biological Engineering, University of Minho, Campus de Gualtar, Braga, Portugal; 2 European Molecular Biology Laboratory, Heidelberg, Germany; 3 Instituto de Tecnologia Química e Biológica António Xavier, Universidade Nova de Lisboa (ITQB-NOVA), Oeiras, Portugal; The Pennsylvania State University, UNITED STATES

## Abstract

Essential metabolic reactions are shaping constituents of metabolic networks, enabling viable and distinct phenotypes across diverse life forms. Here we analyse and compare modelling predictions of essential metabolic functions with experimental data and thereby identify core metabolic pathways in prokaryotes. Simulations of 15 manually curated genome-scale metabolic models were integrated with 36 large-scale gene essentiality datasets encompassing a wide variety of species of bacteria and archaea. Conservation of metabolic genes was estimated by analysing 79 representative genomes from all the branches of the prokaryotic tree of life. We find that essentiality patterns reflect phylogenetic relations both for modelling and experimental data, which correlate highly at the pathway level. Genes that are essential for several species tend to be highly conserved as opposed to non-essential genes which may be conserved or not. The tRNA-charging module is highlighted as ancestral and with high centrality in the networks, followed closely by cofactor metabolism, pointing to an early information processing system supplied by organic cofactors. The results, which point to model improvements and also indicate faults in the experimental data, should be relevant to the study of centrality in metabolic networks and ancient metabolism but also to metabolic engineering with prokaryotes.

## Author summary

If we tried to list every known chemical reaction within an organism–human, plant or even bacteria–we would get quite a long and confusing read. But when this information is represented in so-called genome-scale metabolic networks, we have the means to access computationally each of those reactions and their interconnections. Some parts of the network have alternatives, while others are unique and therefore can be essential for growth. Here, we simulate growth and compare essential reactions and genes for the simplest type of unicellular species–prokaryotes–to understand which parts of their metabolism are universally essential and potentially ancestral. We show that similar patterns of essential reactions echo phylogenetic relationships (this makes sense, as the genome provides the building plan for the enzymes that perform those reactions). Our computational predictions correlate strongly with experimental essentiality data. Finally, we show that a crucial step of protein synthesis (tRNA charging) and the synthesis and transformation of small molecules that enzymes require (cofactors) are the most essential and conserved parts of metabolism in prokaryotes. Our results are a step further in understanding the biology and evolution of prokaryotes but can also be relevant in applied studies including metabolic engineering and antibiotic design.

## Introduction

Prokaryotes are the simplest contemporary life forms known, and nevertheless are characterized by an immense complexity. The debate on the features of such complexity and its breadth in the primordial life forms has been around for years [1–3], and was furthermore expanded and detailed since the advent of systems biology [4–6]. The study of essential genes has been a crucial contribution for detangling this complexity, relating some proteins with cell viability in specific conditions [7] and others with cell viability in apparently all conditions [8,9]. Genome-wide essentiality studies based on collections of targeted mutants or generated by random mutagenesis have been conducted for a number of species, aiming mainly at antibiotic design or identifying industrially relevant targets [10–15]. These data have been made available in databases such as the Online Gene Essentiality Database (OGEE) [16] and the database of essential genes (DEG) [17] but their comparative and integrative analysis, although having already provided relevant insights, is still emerging. Early work comparing genome-scale essentiality data of *Mycoplasma genitalium*, *Haemophilus influenza*, *Bacillus subtilis* and *Escherichia coli* found that it is essentiality, not expressiveness, that drives gene strand bias [18]. Later, a critical review of genome-scale essentiality datasets conducted a preliminary analysis integrating six assays corresponding to four different species [19]. Functional differences were highlighted, as the smaller number of essential genes in flavin synthesis in *B*. *subtilis*, a species known to have an active riboflavin salvage capability. The authors of the DEG database have also conducted a pair of integrative analyses on large-scale essentiality data. The first concluded that there are less essential genes inside than outside genomic islands, and some of those are related with virulence [20]. The second study [21] added to a previous finding based only on *E*. *coli* essentiality data where it was proposed that essential genes are more evolutionarily conserved than non-essential genes [22]. Luo and others used the same type of analysis, based on synonymous and non-synonymous substitution rates for 23 bacterial species, to corroborate this finding [21]. The study indicates that the most evolutionarily conserved COG categories of essential genes are carbohydrate transport and metabolism; coenzyme transport and metabolism; transcription; translation, ribosomal structure and biogenesis; lipid transport and metabolism, and replication, recombination and repair.

Genome-scale metabolic models (GSMs) are large curated repositories of metabolic data for individual species that expand possibilities of analysis of cellular physiology [23]. Apart from improving or suggesting new functional annotations by reconstructing whole pathways [24], GSMs can be used for calculating metabolic fluxes that permit the prediction of, among others, lethal phenotypes [25]. Multi-species analysis of this type of phenotype predictions with different manually curated models has been scarce [26], in part impaired by the poor knowledge basis for other species than the usual model organisms, but also by the deficient use of standards in building such models [27].

Comparative genomics is commonly used to find core essential genes for several species, and being based on the key evolutionary notion of orthology, to infer genes present in common ancestors [28]. Evolutionary parsimony indicates that genes present in a set of species have been vertically inherited from a common ancestor. Horizontal Gene Transfer (HGT) might have played a role even before the divergence of the three main domains [29], but when a gene is present in all or most species of a phylogenetic branch, the most parsimonious scenario is vertical inheritance.

In parallel with genetic comparisons, genome-scale metabolic models allow for functional comparisons at the level of metabolic capacities (reactions). Building up on this methodological advantage, in this study, 36 experimental genome-scale essentiality assays were integrated with simulation results from 15 genome-scale metabolic models to reveal common patterns of essentiality. To this analysis the screening of full genome sequences of 79 prokaryotic species was added in order to find core conserved functions in prokaryotic biology. It is expected that this knowledge on the minimal metabolic functions of prokaryotic cells can not only help untangling the fundamental complexity of cellular systems but also, by building up on the concept of orthogonalization of metabolic modules [30], here analysed in the form of metabolic subsystems (pathways), improve future engineering approaches that use this type of organisms.

## Methods

### Genome-scale metabolic models used in essentiality predictions

For all essentiality predictions performed in this study, 15 genome-scale metabolic models were chosen based on curation, validation, and comparability of the nomenclature of metabolites and reactions. These comprise 7 prokaryotic phyla, including one archaea. Ten of these models include more than 20% of the total of ORFs of the corresponding species. Table 1 summarizes the details on the models used, including species name, model ID, content and references.

**Table 1:** Details on models and corresponding species used in *in silico* essentiality simulations.

Phylum	Species	Model ID	# Reactions	# Metabolites	% ORFs	Reference
Firmicutes	*Bacillus subtilis*	iYO844	1020	988	21%	[31]
	*Clostridium beijerinckii NCIMB 8052*	iCB925	938	881	18%	[32]
	*Staphylococcus aureus N315*	iSB619	641	571	24%	[33]
Proteobacteria	*Escherichia coli K12*	iAF1260	2077	1039	29%	[34]
	*Escherichia coli W (ATCC9637)*	iCA1273	2477	1111	27%	[35]
	*Helicobacter pylori 16695*	iIT341	476	485	21%	[36]
	*Klebsiella pneumoniae MGH 78578*	iYL1228	1970	1658	24%	[37]
	*Pseudomonas putida KT2440*	iJN746	950	911	14%	[38]
	*Salmonella typhimurium LT2*	STM_v1.0	2201	1119	28%	[39]
	*Shewanella oneidensis MR-1*	iSO783	774	634	15%	[40]
Actinobacteria	*Mycobacterium tuberculosis H37Rv*	iNJ661	939	828	15%	[41]
Chloroflexi	*Dehalococcoides ethenogenes*	iAI549	518	549	27%	[42]
Cyanobacteria	*Synechocystis sp*. *PCC6803*	iJN678	863	795	21%	[43]
Thermotogales	*Thermotoga maritima MSB8*	(None)	562	503	25%	[44]
Euryarchaeota	*Methanosarcina barkeri str*. *Fusaro*	iAF692	476	485	14%	[45]

### Environmental conditions for simulations

All GSMs were collected in SBML format and then parsed to model an environmental condition corresponding to rich media: all original exchange reactions in the model were set to a maximum uptake limit of -20 mmol gDW^-1^ h^-1^ to allow for the import of all transported metabolites (including oxygen, whenever possible).

### *In silico*, single deletion of metabolic reactions

Flux Balance Analysis (FBA) [46,47] was used to predict the essentiality of each metabolic reaction in all models. A threshold of 10% of the flux through the biomass reaction compared to the wild type was set as the limit to define an essential metabolic reaction. All modelling procedures were implemented in C++ and solved using IBM ILOG CPLEX solver. The Optflux platform [48] was used occasionally to benchmark results.

### Standardizing the nomenclature of essential metabolic reactions

For comparison of the essential reactions calculated for the 15 GSMs, some nomenclature inconsistencies were resolved: standardization of suffixes used in reaction IDs, removal of unnecessary or redundant indications of reversibility and species names allocated to reactions and other redundant tags. Irrelevant and irregular characters such as dashes were filtered out of the nomenclature (the final list of standardized reaction ids for reactions essential at least once is provided in the S1 File).

### Experimental data and subsystem mapping

Large-scale experimental data on gene essentiality were collected from two databases, OGEE [16] and DEG [17]. The content of the databases was compared and DEG was chosen for it was considerably larger, including wider and clearer annotation metadata for 36 prokaryotic datasets (Table 2). Genes were mapped to the subsystems present in the latest *Escherichia coli* genome-scale metabolic model [49] using the DEG integrated nomenclature system of gene identifiers. All essential reactions obtained after GSMs analysis were also mapped according to this updated list of subsystems, using their standardized nomenclature (see above; S2 File).

**Table 2:** Large-scale essentiality assays used in this study, number of essential genes in each and respective original reference of publication.

Species name	Number of essential genes		Reference ^a^
*Acinetobacter baylyi ADP1*		499	[12]
*Bacillus subtilis 168*		271	[50]
*Bacteroides fragilis 638R*		547	[51]
*Bacteroides thetaiotaomicron VPI-5482*		325	[52]
*Burkholderia pseudomallei K96243*		505	[53]
*Burkholderia thailandensis E264*		406	[54]
*Campylobacter jejuni subsp*. *jejuni NCTC 11168 = ATCC 700819*		228	[55]
*Caulobacter crescentus*		480	[56]
*Escherichia coli MG1655 I*		609	[10]
*Escherichia coli MG1655 II*		296	[57]
*Francisella novicida U112*		392	[11]
*Haemophilus influenzae Rd KW20*		642	[58]
*Helicobacter pylori 26695*		323	[59]
*Methanococcus maripaludis S2*		519	[60]
*Mycobacterium tuberculosis H37Rv*		614	[61]
*Mycobacterium tuberculosis H37Rv II*		771	[62]
*Mycobacterium tuberculosis H37Rv III*		687	[63]
*Mycoplasma genitalium G37*		381	[64]
*Mycoplasma pulmonis UAB CTIP*		310	[65]
*Porphyromonas gingivalis ATCC 33277*		463	[66]
*Pseudomonas aeruginosa PAO1*		117	[67]
*Pseudomonas aeruginosa UCBPP-PA14*		335	[68]
*Salmonella enterica serovar Typhi*		353	[14]
*Salmonella enterica serovar Typhi Ty2*		358	[69]
*Salmonella enterica serovar Typhimurium SL1344*		353	[69]
*Salmonella enterica subsp*. *enterica serovar Typhimurium str*. *14028S*		105	[70]
*Salmonella typhimurium LT2*		230	[71]
*Shewanella oneidensis MR-1*		403	[72]
*Sphingomonas wittichii RW1*		535	[73]
*Staphylococcus aureus N315*		302	[74]
*Staphylococcus aureus NCTC 8325*		351	[15]
*Streptococcus pneumoniae*		244	[75]
*Streptococcus pyogenes MGAS5448*		227	[76]
*Streptococcus pyogenes NZ131*		241	[76]
*Streptococcus sanguinis*		218	[77]
*Vibrio cholerae N16961*		779	[13]

^a^ The corresponding annotated data was obtained from the DEG database [17].

### Analysis of genetic conservation

To analyse the conservation and infer ancestry of all the genes annotated in metabolic subsystems of GSMs, a local protein blast was performed against representative genomes of all the 35 prokaryotic phyla with at least one fully sequenced quality genome in the NCBI genome database (version June 2015). For this task, translated genomes were selected and downloaded for 53 unique species of prokaryotes for which an available GSM could be found; to these, 26 representative genomes for phyla not modelled with GSMs were added. This totalled in 79 translated genomes representing the 35 fully sequenced phyla in the current tree of life of prokaryotes (see S2 Fig, built with iTOL v. 4.2.1 [78] which can be reproduced in iTOL with the corresponding NCBI taxonomy). All of the protein-encoding genes of *E*. *coli* K12 (RefSeq genome NC_000913.3) were used as queries and annotated to the subsystems of the latest *E*. *coli* model [49]. The threshold e-value considered was 1e-10. All the procedures were implemented using the Biopython package [79].

### Numerical and statistical analysis of essentiality and conservation

For assessing the conservation of essential reactions and essential genes in each metabolic subsystem, the weighted sum of essentiality (**W**) was calculated for each subsystem **m**, as:

$$W_{m}=\sum_{i=1}^{t}n_{i}*i$$ (1)

***n_i_*** being the number of reactions or genes of that subsystem essential in ***i*** models or datasets, where **t** is the total of models or datasets, 15 and 36 respectively.

The score sum of experimental essentiality for each individual gene *S_g_* was calculated as the number of times a gene was found essential (E) minus the number of times it was found not essential (N) in all experimental assays datasets:

$$S_{g}=E-N$$ (2)

All statistical analyses were performed using R (statistical software, version 3.1). Hierarchical clustering was performed using the ‘pvclust’ R package [80] with binary distance as the dissimilarity metric and Ward 1 method as the linkage criterion. Pvclust was also used for assessing uncertainty by calculating approximately unbiased p-values via multiscale bootstrap resampling. Both the Fisher and Kolmogorov Smirnov tests were performed in R as well with the corresponding default parameters.

## Results

### Single-reaction and single-gene essentiality data reflect phylogenetic patterns

**Fig 1:**
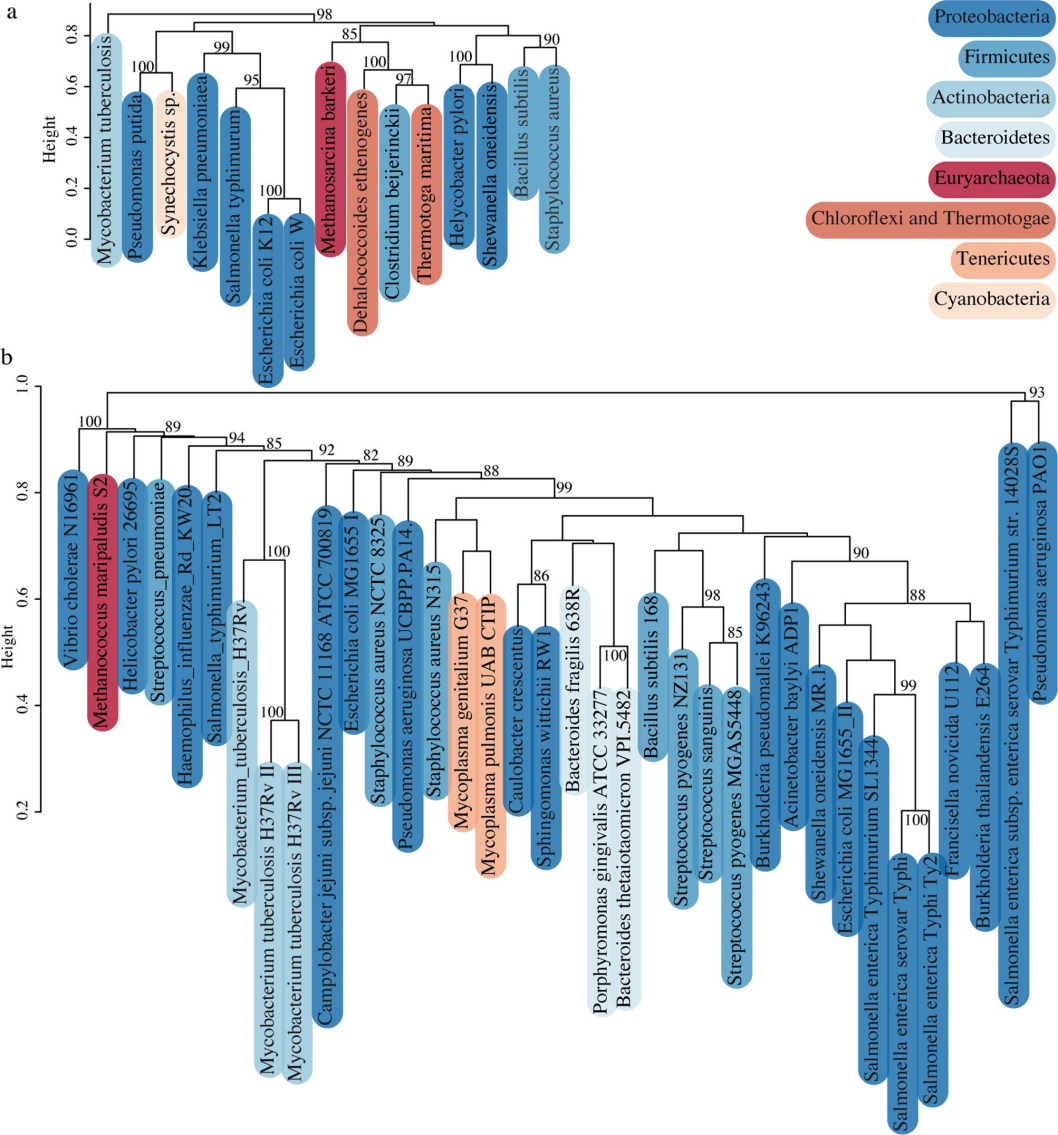
**Clustering of (A) simulated and (B) experimental genome-scale essentialities of prokaryotes.** Clusters show approximately unbiased p-values greater than 80% calculated by multiscale bootstrap re-sampling with 1000 replicas (see Methods for details). Phyla are coloured according to the taxonomical representation of the prokaryotic tree of life built with iTOL [78] (tree in S2 Fig).

To analyse the validity of the essentiality results on a large scale, the different models were clustered based on single-reaction essentiality predictions and the different datasets available on DEG [17] were clustered based on the content of essential genes (Fig 1).

In the case of the simulated essentiality (Fig 1A), strongly supported clusters (more than 80% of 1000 bootstrap replicas) are phylogenetically consistent at the level of the phylum–they cluster in a statistically significant manner with at least one sister species–with the exception of the models of *C*. *beijerinckii* and *P*. *putida*. *H*. *pylori* and *S*. *oneidensis* show up in the same cluster, but not together with the rest of the Proteobacteria, but the two high-level clusters (that exclude *M*. *tuberculosis* with a p-value of 98%) are not statistically supported (p-values of 58 and 62% for the left and right cluster respectively). The lower number of available exchange reactions in the models of *H*. *pylori*, *S*. *oneidensis* and *P*. *putida* (74, 95 and 89 respectively) compared with the models for other Proteobacteria (*K*. *pneumoniae*, *E*. *coli* K12, *S*. *typhimurium* and *E*. *coli W* with 289, 299, 305 and 310 respectively) points to a justification for these results, as less exchanges cause more reactions in the network to be essential. *C*. *beijerinckii’*s model is also very restricted with regards to exchange reactions, with only 19 metabolic drains available.

Regarding the experimental data (Fig 1B), there is also a pattern of clustering taxonomically related species. One well-supported phylogenetic cluster is that of several gamma and beta-proteobacteria, including *Acinetobacter baylyi*, dataset II of *E*. *coli* K12, three Salmonellas, one *Shewanella* and one *Francisella*. Others are the cluster of Bacteroidetes, the cluster with all three datasets of *M*. *tuberculosis* and the cluster of the alpha-proteobacteria, *Sphingomonas* and *Caulobacter*. The datasets of *Pseudomonas aeruginosa PAO1* and *Salmonella enterica subsp*. *Enterica serovar Typhimurium str*. 14028S cluster together likely due to not being saturated genome-wide gene-essentiality screens (these datasets are considerably smaller, with 117 and 105 genes respectively–see Table 2 for context). Surprisingly, Firmicutes are spread all across the tree. Also, both *E*. *coli* sets are very distant from each other. Although they were performed under rich media conditions, one yielded 609 essential genes and the other only 296 (Table 2). This difference is likely due to the use of different technologies to perform the large-scale assays, the first being random mutagenesis and the screening of mixed populations, and the second the screening of libraries of targeted mutants, as reviewed in [19].

### Cofactor metabolism is the most essential subsystem both in simulated and experimental data

**Fig 2:**
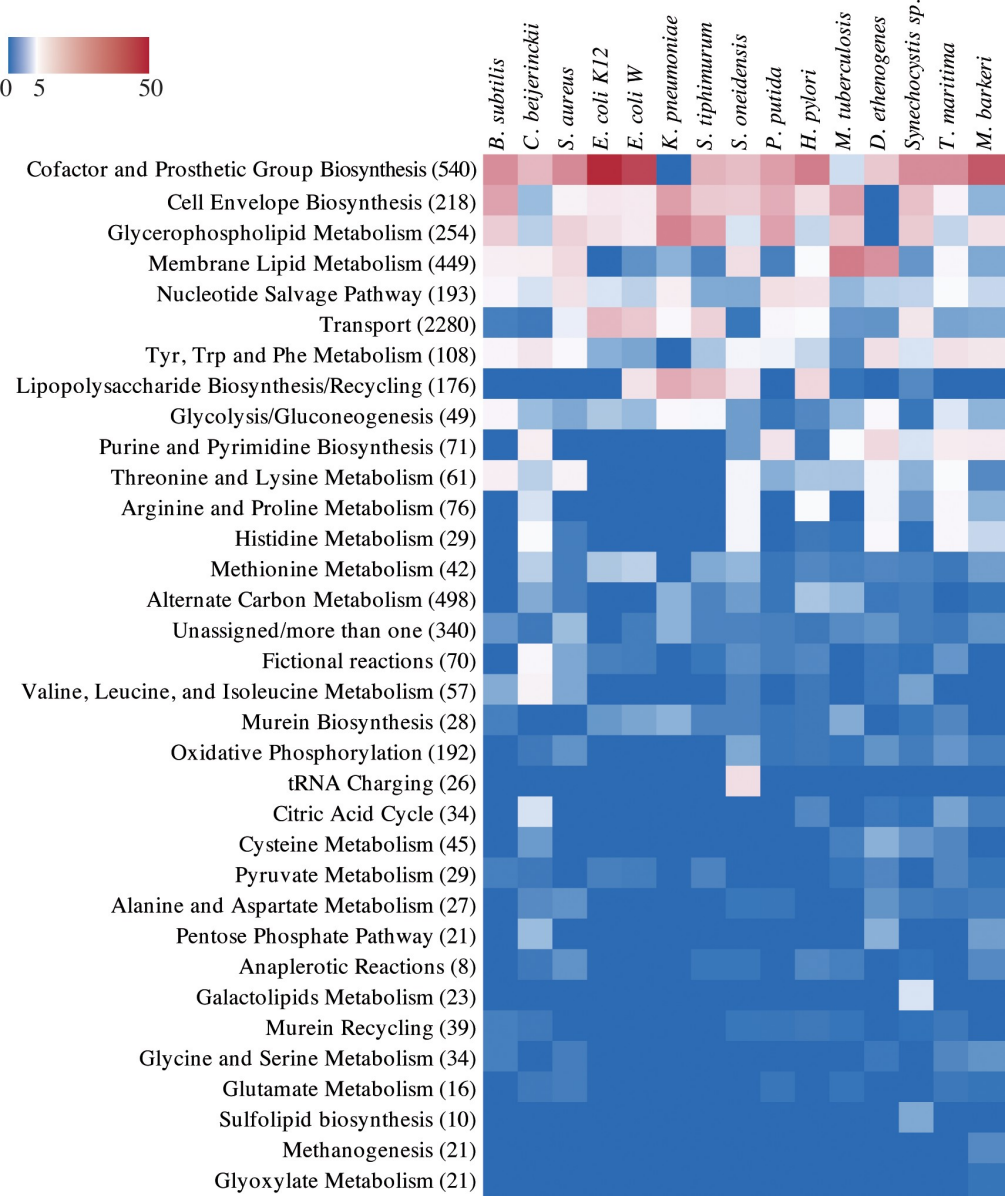
Essentiality for biomass production of each metabolic subsystem in 15 genome-scale manually curated metabolic models. The colour bar represents the ratio of essential reactions (in that subsystem) to the total of essential reactions in each model (0%—blue; 5%—white; 50%—red). In parenthesis next to the subsystem name is the total of reactions in that subsystem (for all models).

Next, all the essential reactions calculated for the 15 GSMs were mapped to the corresponding metabolic subsystem (see Methods; S1 and S2 Files). Different models show different proportions of essential reactions for each subsystem (Fig 2).

For the majority of the models, the most essential subsystem is that of cofactor and prosthetic group biosynthesis. Nearly 48% of the essential reactions in the simulations with the GSM of *E*. *coli* K12–54 reactions–were related with this subsystem (Fig 2). Several of the predicted essential reactions were confirmed to be essential steps in the biosynthesis of the active forms of cofactors that cannot be directly uptaken, e.g. dihydrofolate synthase and dihydrofolate reductase for the biosynthesis of tetrahydrofolate and derivatives [81], NAD kinase for obtaining NADP [82] and riboflavin synthase in some species [83]. For *M*. *tuberculosis*, *D*. *ethenogenes*, *S*. *typhimurium and K*. *pneumoniae* the most represented subsystems were glycerophospholipid or membrane lipids metabolism (30.5, 25.1, 21.8 and 29.2% of all essential reactions, respectively). Discrepancies regarding results for each individual model are not only related with differences in the metabolic network but are also dependent on the formulations of the biomass equation (e.g. the biomass equation in *Klebsiella pneumoniae*’s model lacks cofactors) [84].

**Fig 3:**
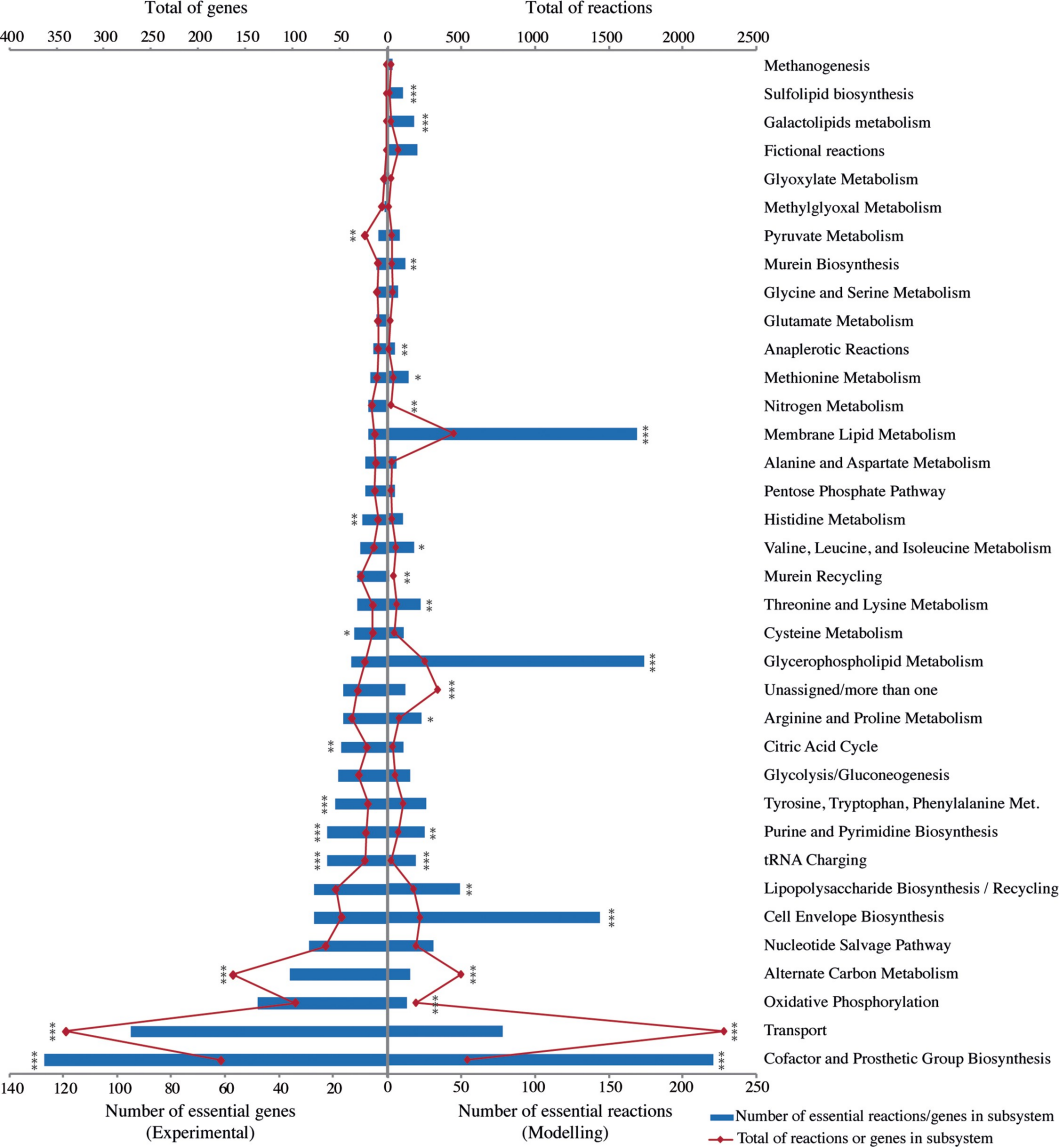
Number of essential reactions predicted by genome-scale metabolic models and essential genes in genome-scale experimental assays for different metabolic subsystems. Each reaction and gene were counted only once, even if present (or present and essential) in more than one model or experimental dataset. Single, double and triple asterisks indicate p-values less than 0.05, 0.01 and 0.0001, respectively, after a Fisher's exact test for count data testing independence of the number of essential vs the number of non-essential mapped genes and reactions.

To validate the predictions of essentiality of metabolic subsystems obtained with GSMs, each experimentally essential gene in DEG was annotated according to its function, using the same system used in GSM’s. This system (see Methods) covered more annotations when compared with COG annotations (1363 essential metabolic genes annotated in total, when compared with a total of 906 unique metabolic COGs–S1 Fig). Moreover, this curated dataset could be directly compared to the modelling results and included some genes annotated in the “General function prediction only” COG category. After annotation, all unique essential genes were identified and the same was done for essential reactions in the models. Both the total number of unique essential reactions (modelling) or genes (experimental) varies significantly between subsystems (Fig 3**)**. The total of reactions and genes in each subsystem (for both modelling and experimental data, respectively) also varies significantly. To test for independence of the totals of essentials from the sizes of the subsystems, we performed a Fisher’s exact test, and for the majority of subsystems the null hypothesis of dependence was rejected (p-value less than 0.05).

In Fig 3 the subsystem of Cofactor and Prosthetic Groups biosynthesis appears isolated with the maximum number of unique essential enzymes both in experimental and modelling data. Three subsystems show a striking difference in the ranking between experimental and modelling data, being more represented in the latter, all related with membrane and cell wall metabolism. Several justifications can be raised for this difference. First and foremost, often in those subsystems the number of different reactions that can be encoded by the same gene is high [85], (thus there will be several essential reactions for each essential gene). For instance, in the model of *Synechocystis* there are twelve essential reactions related with fatty acid biosynthesis all encoded by the same gene, fabZ (sll1605 in the model), a gene that is essential in rich medium experimentally, but in that case, counted only once. Similarly, all of the twenty essential fatty acid synthase reactions in the model of *M*. *tuberculosis* are encoded by only 3 different genes: Rv1663 and Rv1662 or Rv2940c. There might also be some lack of integration in nomenclature of the reactions in these subsystems in the different GSMs that should not contribute significantly as all models follow the same nomenclature scheme, which was still manually curated for the essential reactions.

### Conservation of essentiality validates predictions by GSMs with experimental data and pinpoints specific model gaps

To further explore essentiality at the level of genes and reactions, the conservation of essentiality across models and experimental datasets was analysed for each reaction and gene in each metabolic subsystem. Strikingly, no reaction was essential in all the models analysed. Three reactions annotated within aromatic amino acids metabolism (shikimate kinase, 3-phosphoshikimate 1-carboxyvinyltransferase and chorismate synthase) were essential in all models except for *K*. *pneumoniae*. Although there are differences in the models regarding the capacity to uptake aromatic amino acids, this does not justify the observed differences in terms of essentiality. The notable difference between the model of *K*. *pneumoniae* and the others is that it lacks cofactors and prosthetic groups in its biomass equation. Those three reactions correspond to the three last steps in the synthesis of chorismate, which is part of the shikimate pathway, which connects central metabolism with aromatic amino acids metabolism. However, this pathway is also the route taken to synthesize several other compounds in the cell, including quinones and folates [86], which are not present in the biomass equation of *Klebsiella*, which is likely the cause for the difference in the results as discussed in [84] and above.

Reactions essential in several models annotated within the cofactor and prosthetic group biosynthesis subsystem are dihydrofolate synthase, essential in 13 out of the 15 models, and dihydrofolate reductase and NAD kinase, both essential in 12 models. These are related with the biosynthesis of folates and the phosphorylation of NAD to produce NADP. Two reactions involved in the salvage pathways of nucleotides–the biosynthesis of GDP and dTTP—were also essential for 14 models. Three reactions related with the biosynthesis of cell wall components are essential in all models except for *B*. *subtilis* and *D*. *ethenogenes*—glucosamine-1-phosphate N-acetyltransferase, phosphoglucosamine mutase and UDP-N-acetylglucosamine 1-carboxyvinyltransferase. Acetyl-CoA carboxylase, related with membrane lipid metabolism, is essential in 12 of the 15 models. One reaction not assigned to any subsystem, the HCO_3_^-^ equilibration reaction, was essential in 11 of all 15 models.

**Fig 4:**
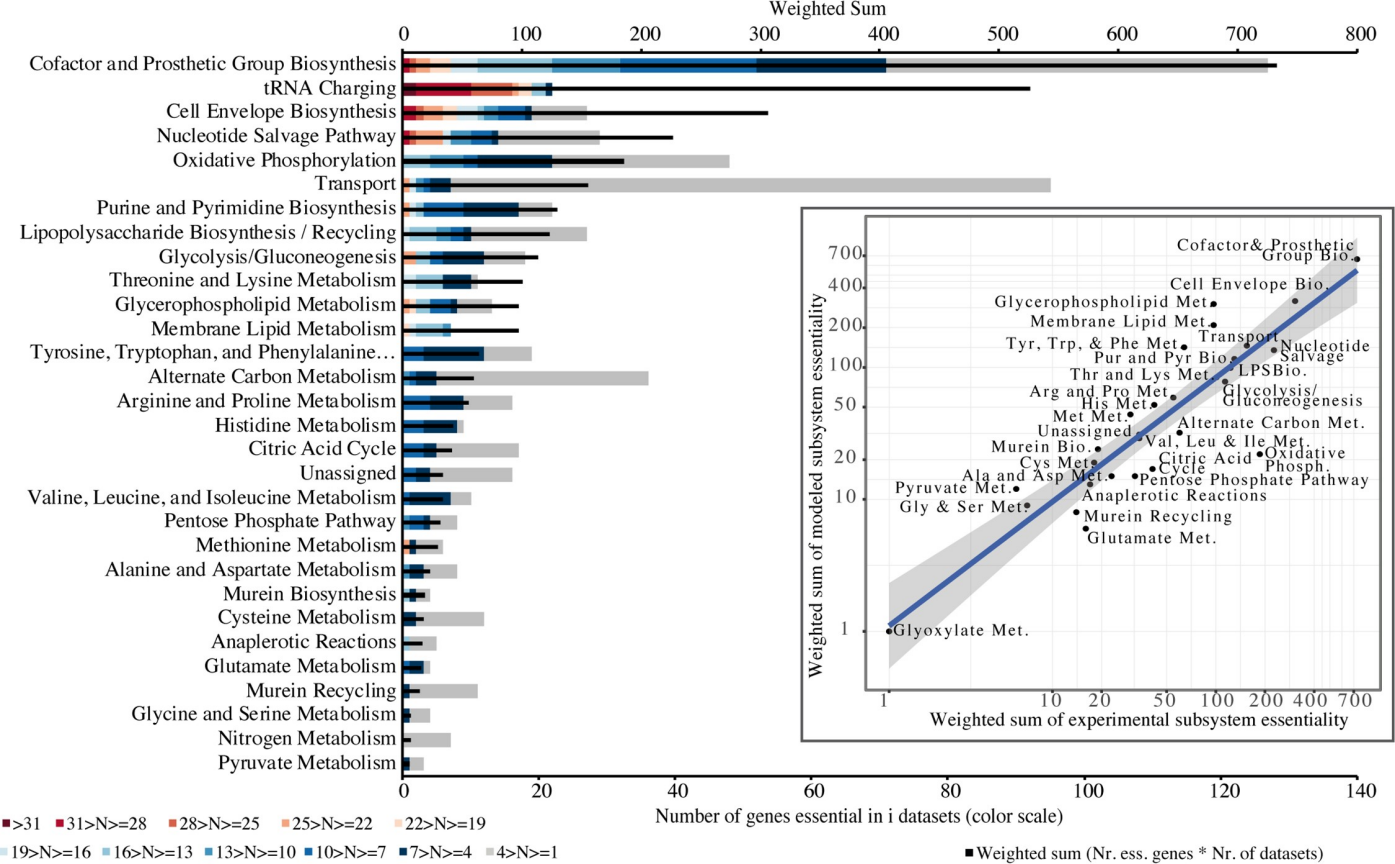
Conservation of essentiality of metabolic subsystems in 36 large-scale gene essentiality datasets and correlation with modelling predictions (inset). Red indicates highest conservation (genes essential in more than 31 datasets) and grey the least (essential in less than 4 datasets); black bar: weighted sum of essential genes given the number of datasets in which they are essential (see Methods). Inset plot: Correlation between modelling and experimental genome-scale essentiality data at subsystem level (adjusted R^2^ of 0.821, Pearson correlation coefficient of 0.909 with p-value 9.65e-14); axes are represented in log scale for visualization purposes only.

To overview the relationship between modelling and experimental results, the conservation of essentiality for each set of results was compared. The inset plot in Fig 4 shows the high correlation obtained between the weighted essentiality for each subsystem between simulated and experimental data. However, there are some differences to be noted. Firstly, regarding the tRNA charging subsystem, it is modelled in only one GSM (*S*. *oneidensis*, Fig 2). This causes this category to appear much more evidently as the second most conserved essential subsystem in experimental data, in contrast with the low result in the simulations of GSMs. It is expected that future GSMs will include this subsystem, but for the sake of comparison of these results, it was excluded from the correlation.

Interestingly, regarding the three highly essential reactions in modelling results related with chorismate biosynthesis, these were not found as significantly essential in experimental data. It is known that in minimal media the knock-out of chorismate synthase (aroC) in *E*. *coli* impairs growth [87]. The non-essentiality of this enzyme in the rich media analysed here indicates that there must be a compound in the media compensating for its absence. It has been shown that, when provided with p-aminobenzoic acid (PABA), para-hydroxybenzoic acid (PHBA) or a combination of a precursor from PABA with a non-biological catalyst, the growth of *E*. *coli* aroC mutant in M9 minimal medium can be rescued [88]. PABA and its derivatives cannot be uptaken in the genome-scale model of *Escherichia coli* or any other of the working set. Transporters for these compounds or others that might compensate in rich media for lethal phenotypes in minimal media remain to be integrated in the genome-scale metabolic models and further explored.

Again, the subsystem of cofactor and prosthetic groups metabolism has the highest number of reactions appearing as essential in more datasets in experimental data, in accordance with modelling data. Dihydrofolate synthase and reductase (highest ranking in modelling) are essential in 11 and 25 experimental datasets, respectively, indicating that either several organisms can overcome the lack of both enzymes by intermediate pathways not yet modelled, or that the experimental assays have produced false negatives (see the differences between the sizes of the two experimental assays for *E*. *coli* in Table 2 discussed above; a special case, dihydrofolate synthase, is further explored in the Discussion). NAD kinase appears as highly essential, also in accordance with simulations, in 24 datasets. Several other genes encoding for enzymes essential for the biosynthesis of cofactors are highly essential for cell viability experimentally, even in rich media (eg. nadE for NAD; coaD and coaE for coenzyme A; hemC for tetrapyrroles; dxr for isoprenoids). Cell envelope biosynthesis genes follow as the third most conserved essential functional module, in accordance with the modelling results as well.

### Core conserved metabolic subsystems

**Fig 5:**
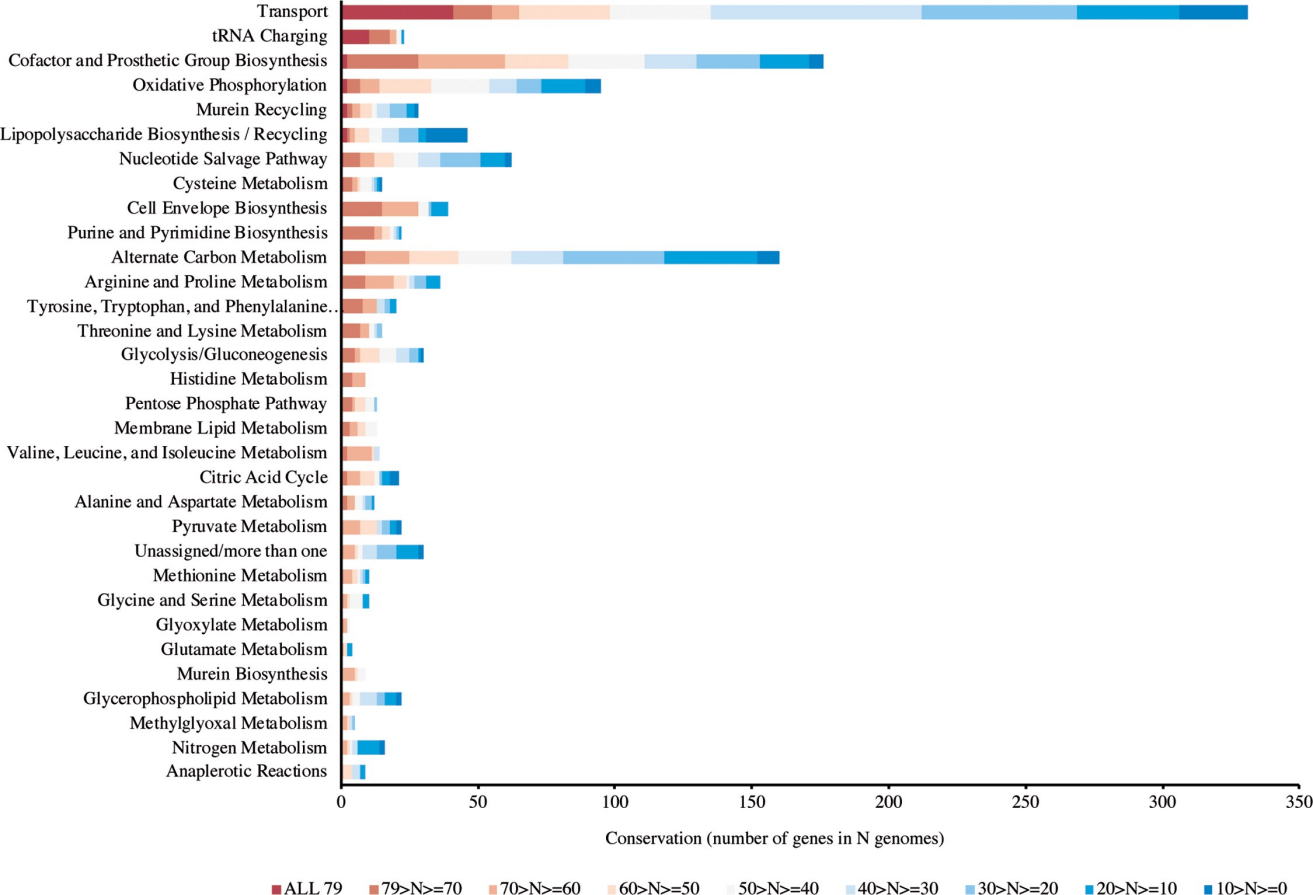
Conservation of metabolic subsystems in genomes of all prokaryotic phyla with at least one fully sequenced genome. Dark red indicates the highest conservation (genes that are present in all 79 genomes accessed) and dark blue the least (present in less than 10 genomes).

Based on the premises of evolutionary parsimony and orthology [28], we proceeded to the analysis at a large scale of the conservation of metabolic genes in the prokaryotic tree of life to infer potential ancestral metabolic functions. Seventy-nine genomes were assayed, representing all the known prokaryotic phyla with a fully sequenced genome (see Methods for details). A phylogenetic tree with these 79 species is available in S2 Fig. All annotated metabolic genes of *E*. *coli* K12 were used as queries to search the set of genomes for conserved metabolic genes and respective functions. The results on conservation of metabolic genes are summarized in Fig 5.

The metabolic subsystem with more prevalent genes is Transport, followed by the tRNA charging subsystem with 18 nearly universal aminoacyl-tRNA synthetases. It should be noted though that nearly all of the 41 transport genes conserved in all 79 genomes correspond to ABC transporters (S1 Table) with a ubiquitous ATP-binding domain. Two genes involved in oxidative phosphorylation were also conserved in all genomes analysed: atpA and atpD (ATP synthase subunits alpha and beta, respectively). In the subsystem of cofactors and prosthetic group biosynthesis, glutX and sufC were also conserved in all genomes analysed. It should be noted that glutX corresponds to a tRNA charging protein, a glutamyl-tRNA synthetase involved in the biosynthesis of heme, that should have a double annotation; sufC is an atypical cytoplasmic ABC/ATPase, required for the assembly of iron-sulphur clusters [89]. Although the vast majority of genes found conserved in all the genomes analysed correspond to ABC ubiquitous domains, the high conservation (between 70 and 79 genomes) of 214 other genes is still prominent. Twenty-eight of those are classified in the subsystem of cofactor and prosthetic group biosynthesis genes (Fig 5, S2 Table).

### Common essential genes are rarer and prone to be highly conserved, contrarily to common non-essential genes

**Fig 6:**
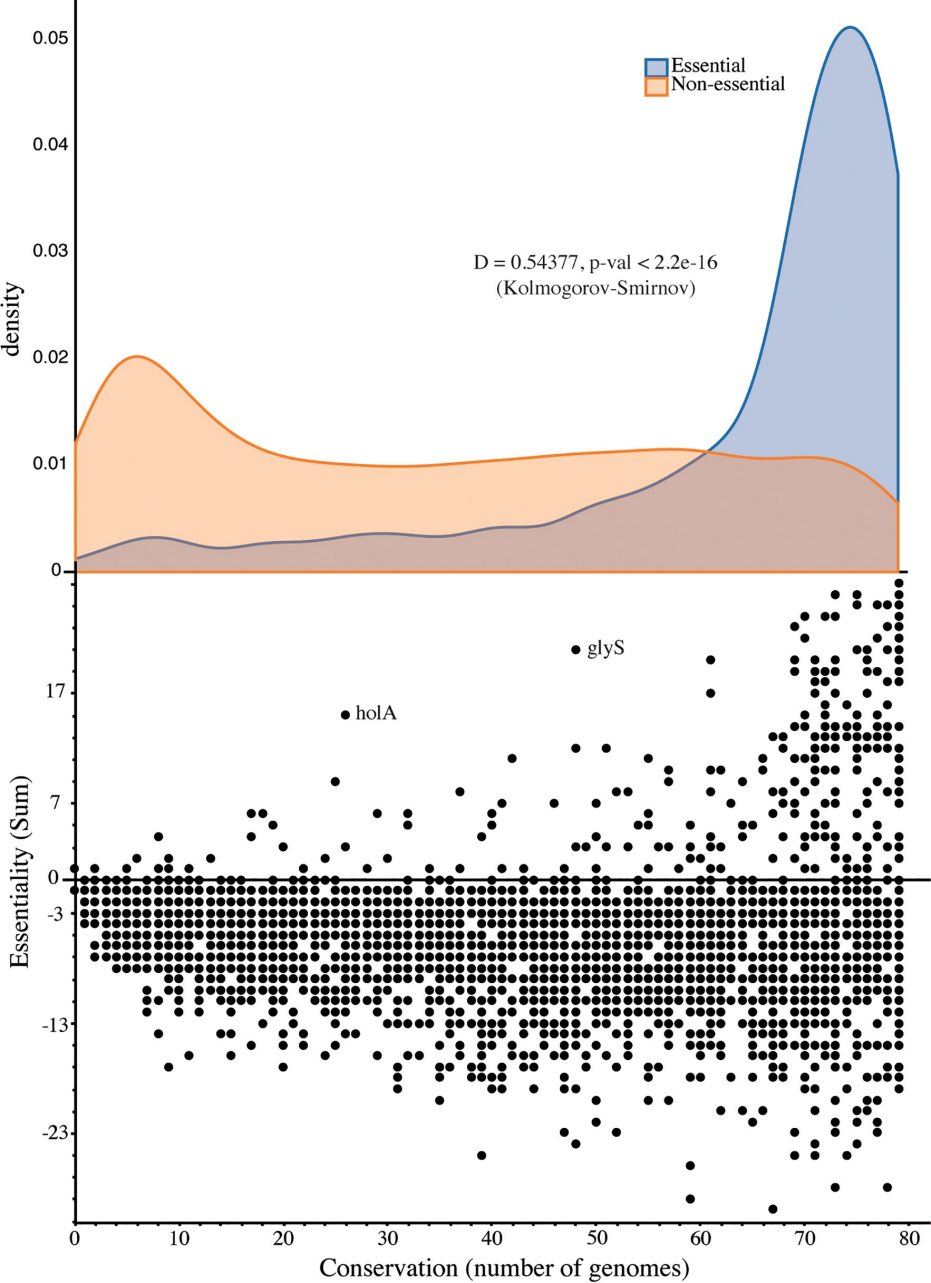
Conservation and essentiality of protein-encoding genes. Conservation is calculated as the number of genomes where a gene is present and essentiality as the number of datasets where a gene is essential minus the datasets where it is non-essential. The full list of genes is given in S3 File. In the bottom panel, all data is plotted, with conservation on the x axis and essentiality on the y axis. The top panel depicts the density distribution of the conservation of genes (on the shared x axis) with a sum of essentiality larger than 0 –blue–and that of the genes with a sum of essentiality equal or smaller than 0 –orange. The results of a Kolmogorov–Smirnov test for the independence of both distributions are shown, D for the value of the test statistic and the p-value of the test.

On a first look, there is no correlation between essentiality (Fig 4) and conservation (Fig 5) at the individual gene level. The same is even more evident in the case of the highly conserved ABC domains in transporters (S1 Table), with the majority (20/33) not being essential in any dataset in DEG. This substantiates the fact that highly conserved genes are not necessarily highly essential, likely due to genetic redundancy (see Discussion). The only subsystem for which the correlation is positive and significant (Pearson coefficient 0.95, p-value 1.07e-06) is Membrane Lipid Metabolism. To assess the unbiased relationship between essentiality and conservation at the individual gene level, we compared data on conservation with the experimental data on essentiality. For this purpose, we calculated a sum score for each gene’s experimental essentiality and compared it with its conservation (Methods, Fig 6). This allows for measuring the level of evidence of essentiality for each gene individually with precision.

In the bottom panel of Fig 6, the vast majority of genes lie on the bottom area of the plot (sum of essentiality below zero), meaning that they show up in more datasets as non-essential than as essential. However, on the top side of the same plot, where the genes with a positive sum of essentiality lie, the clear majority are shifted to the right–meaning that they are highly conserved. There are some interesting outliers, such as glyS–glycine-tRNA ligase–highlighted in Fig 6. This corresponds to one instance in which the monophyly rule is violated: *E*. *coli’s* type is common for most bacteria, but another type is common to some other bacteria, archaea and eukarya [90,91]. Another interesting case is holA, also highlighted, a DNA polymerase delta subunit that is also divergent in its evolutionary history [50,92] but which has been poorly studied. Upon splitting the genes into two sets–those with a positive sum of essentiality and those with a null or negative sum, two very different distributions are obtained, shown in the top panel of Fig 6. A Kolmogorov-Smirnov test states on the independence of both distributions–essential genes are much more likely to be conserved, whereas non-essential genes can or not be highly conserved. The full data on conservation and essentiality sums can be found in S3 File.

## Discussion

The integration done here was, to our knowledge, the first of the kind for a wide variety of phyla of the bacteria and archaea domains, encompassing experimental phenotypic data, results of large-scale computational simulations and sequence data. The experimental genome-scale essentiality data reveal that approximately 25% of prokaryotic essential genes encode for unknown or general functions (categories S and R in S1 Fig), which is a strong warning on the need for experimental studies on the phenotype of these essential proteins for prokaryotic physiology. Moreover, the organisms for which genome-wide essentiality data are available are relatively scarce. While more experimental data are not available, computational models can be valuable tools aiding in the task of decoding prokaryotic metabolism. It is well known that GSMs are limited by the quality of the genome annotations, the formulation of the biomass equation and the pre-defined environmental conditions and other modelling artefacts [27,84], of which the impact in our results we expand below. Here we tried to reduce the impact of these limitations by basing the choice of the models on a large survey of high-quality manually curated models [84] from which 15 balanced, validated, comparable models were chosen, that at the same time represented a wide phylogenetic diversity (Table 1). The analysis of common patterns of essentiality filtered out the unique essential reactions that might represent specific errors related with individual models. In this manner, we can find core and common features in the overlap of all models. Using GSMs is particularly interesting in this sense, as they allow us to step one level above that of genomes and all their redundancy in the form of isozymes, duplications and confusing phylogenetic events as lateral gene transfers and gene losses. Manually curated GSMs are not mere reflections of genomes–they include thorough revisions of the network and addition of necessary reactions that are encoded by unknown genes or spontaneous chemical transformations. These are also assigned subsystems and counted in our results. Last, as recognized by other authors, the predictive power of comparative analysis can be significantly enhanced by using it within the functional context of pathways and subsystems [19].

The GSMs prediction of which metabolic subsystem has more genes that are commonly essential in multiple species–Cofactor and Prosthetic Group Biosynthesis–was accurate (Fig 4). The exception of the experimentally highly essential tRNA-charging functionality that was not reflected in the simulations is due to the hindrance of just one model including this subsystem [40] but it should be fixed if all the models represent appropriately this subsystem in the future. However, the analysis done here, by integrating experimental data with several different models still allowed us to identify this subsystem as highly essential and conserved (Fig 4 and Fig 5). The problem of the unstandardized biomass composition, evidenced by the GSM of *K*. *pneumoniae* not predicting any essential reaction involved in cofactor and prosthetic group biosynthesis due to the fact that none of those compounds is present in the biomass equation (Fig 2) is relevant to the results, a subject that we have already addressed in a recent publication [84]. Due to the incompleteness of the networks, it was not possible to complete the biomass equations with the missing cofactors without an impractical manual editing and curation of most models. However, considering the results obtained here, this incompleteness could readily be identified (Fig 2) and did not impair the prediction of an overwhelming majority of essential reactions related with the subsystem of cofactor and prosthetic group biosynthesis (Fig 4)–the overlap of all other models and the experimental data reveals the conserved essentiality of this subsystem.

The comparison of the modelling and experimental results can help raise specific hypotheses and directions for more detailed investigation, as discussed above for the essentiality of chorismate synthase in GSMs. In the analysed experimental datasets obtained in rich media, chorismate synthase is not essential, but is has been shown that in minimal media in *E*. *coli* the knock-out of its gene impairs growth [87] that can be restored with p-aminobenzoic acid (PABA) or derivatives [88]. The transporters for these compounds should be added to the models, and we expect that a curated analysis of experimental studies of auxotrophies in the literature can point several more additions to GSMs that will improve predictions at the gene-level. Moreover, these results point also to the necessity of performing more often essentiality experiments in defined media. On the other direction, genome-scale models can also indicate improvements for experimental assays. At the moment of writing of this manuscript, a new genome-wide screen for *Bacillus subtilis* was published [93], where folC (dihydrofolate synthase) was found to be essential, confirming our modelling results and contradicting the previous experimental results for *B*. *subtilis* that were used here [50].

The analysis of conservation of metabolic genes here was the first performed using a manually curated annotation system for metabolic pathways and subsystems, with the most complete genome-scale metabolic model of a prokaryote to date [49]. Regarding inferences on ancestry, there are some limitations to our approach. We chose to use a single e-value threshold in a local BLAST–this might be a lax threshold, but at the same time it allows us to recover potential very ancient homologs, tracing back all the way to the Last Universal Common Ancestor (LUCA), and not to incur in debates about in and out-paralogs. Moreover, we used only a sample of prokaryotic genomes– 79 –although we made sure to include representatives of all sequenced phyla to date (S2 Fig), and we took a stringent threshold to indicate and not affirm ancestry (at least 70 out of 79 genomes). Looking at the conservation of subsystems (Fig 5) also allows us to overcome the phylogenetic distribution bias. On another note, looking only for universal genes as markers of ancestry can be a limited approach, due to the phenomena of gene loss and lateral gene transfer–ideally, phylogenetic trees should be built for all genes. A recent study used an innovative and large-scale approach to infer on the genome of LUCA, building all trees for protein families based on 1981 prokaryotic genomes [94]. Interestingly, although using a completely alternative approach, the study also concluded on tRNA charging and cofactor metabolism as being ancient subsystems. These findings corroborate that the genes identified here as present in all genomes of all representative phyla are most likely genes present in the last common ancestor [28]. Overall, our results of high conservation of the tRNA charging system, Transport and Oxidative Phosphorylation point to a last common ancestor metabolic network of prokaryotes where most of the nutrients were uptaked with nonspecific transporters at the expense of ATP and in which tRNA charging was already present. The results also suggest that the catalytic role of cofactors and prosthetic groups was a coin highly sought for in early prebiotic systems still maintained today, as this is the most conserved metabolic subsystem after transport and tRNA charging. It is highly likely that genes encoding for enzymes aiding in cofactor biosynthesis were selected for early in primordial evolution, as was suggested elsewhere for the origin of anabolic pathways in prebiotic systems [95].

This work expanded considerably on previous related studies regarding the relationship between gene conservation and essentiality in width and depth. The demonstration that essential genes are more evolutionary conserved that non-essential [22], corroborated later with more datasets [21], used the ratio of non-synonymous substitutions to synonymous substitutions in the genomes to estimate conservation (Ka/Ks). Here, 36 experimentally essential datasets were used, that included one Archaea (Table 2). The conservation was analysed by looking at the presence of each gene in 79 genomes that were manually selected to represent all the phyla with one fully-sequenced genome in the prokaryotic tree of life. Because each gene might be essential in some datasets in DEG, non-essential in others and not assayed in yet others, instead of analysing essential genes separately from non-essential as in the two aforementioned studies, we used a measure of essentiality for each gene (sum of essentiality) that takes into account the datasets where it shows up as essential and those where it is non-essential (Fig 6). The results show that genes with a positive sum of essentiality (more datasets showing essential than non-essential) are much scarcer than those with a negative sum; however, it is much more likely that they are highly conserved. For genes with a negative sum of essentiality, there is no tendency for high or low conservation, with a uniform distribution of these genes for all the values of conservation (corroborating results by Fang et al. [92]). We also expanded on previous studies [21,22] by integrating *in silico* simulations and functional assessment of the data, with the conclusion that with the exception of the tRNA charging subsystem, the majority of highly conserved genes related with transport and cofactor biosynthesis are not highly essential (Fig 6, S1 and S2 Tables). These two subsystems show low single-gene essentiality most likely due to metabolic redundancy caused by known alternative metabolic routes (for which multiple knock-outs ought to be performed to test for subsystem-level essentiality) complemented with enzymatic activities not yet known (supported by the percentage of genes with general function prediction only S1 Fig) that might also include promiscuous enzymes [96]. The remarkable redundancy of metabolic networks is reflected in the resilience and robustness of prokaryotic life for the billions of years that it has inhabited Earth.

## Supporting information

S1 Fig COG functional categories for prokaryotic essential genes in DEG. COG metabolic functional categories are less detailed than those used in the annotation of metabolic models: both the “Energy production and Conversion” and “Amino acid transport and metabolism” functional categories encompass several of those that are detailed within GSMs, except for the “Transport” category lumped in GSMs, and distributed for each of the major biomolecules in the COG system. Misleading COG annotations included thiO, an essential gene for the biosynthesis of thiamine diphosphate annotated in category E (Amino acid transport and metabolism) and csd, a cysteine desulfurase, essential in 7 datasets and involved in the formation of Fe-S clusters, also annotated in category E.
(PDF)

S2 Fig Phylogenetic reference tree for species used in the analysis of conservation of essential genes (based on NCBI taxonomy). (PDF)

S1 Table Ubiquitous transporter genes. The list includes genes conserved in all 79 prokaryotic genomes analysed. Essentiality is given as the number of datasets in DEG (out of 36) in which each gene is essential. The description is that of the corresponding annotated ORF in the genome of *E. coli* K12.
(PDF)

S2 Table Highly conserved cofactor biosynthesis genes in prokaryotic genomes. Essentiality is given as the number of datasets in DEG (out of 36) in which each gene is essential. Conservation is given as the percentage of the 79 genomes where a significant homolog for this gene was found. The description is that of the corresponding annotated ORF in the genome of *E. coli* K12. Biosynthesized cofactors were manually retrieved from the detailed information available in the Metacyc database.
(PDF)

S1 File Essential reactions in metabolic models. Presence-absence matrix of essential reactions in the 15 GSMs used.
(XLSX)

S2 File Translation of metabolic subsystems of GSMs to a standardized nomenclature. Lists of original subsystem classifications and corresponding translation for all models and all reactions.
(XLSX)

S3 File Essentiality and conservation scores. List of genes with corresponding scores of essentiality and conservation.
(XLSX)
