# Supplementary material for: Metabolic models and gene essentiality data reveal essential and conserved metabolism in prokaryotes
Source: PLoS Comput Biol. 2018 Nov 16;14(11):e1006556. doi: 10.1371/journal.pcbi.1006556 (PMC6283598; doi:10.1371/journal.pcbi.1006556)
Supplement: S1 Fig — COG metabolic functional categories are less detailed than those used in the annotation of metabolic models: both the “Energy production and Conversion” and “Amino acid transport and metabolism” functional categories encompass several of those that are detailed within GSMs, except for the “Transport” category lumped in GSMs, and distributed for each of the major biomolecules in the COG system. Misleading COG annotations included thiO, an essential gene for the biosynthesis of thiamine diphosphate annotated in category E (Amino acid transport and metabolism) and csd, a cysteine desulfurase, essential in 7 datasets and involved in the formation of Fe-S clusters, also annotated in category E. (PDF) [file pcbi.1006556.s001.pdf]

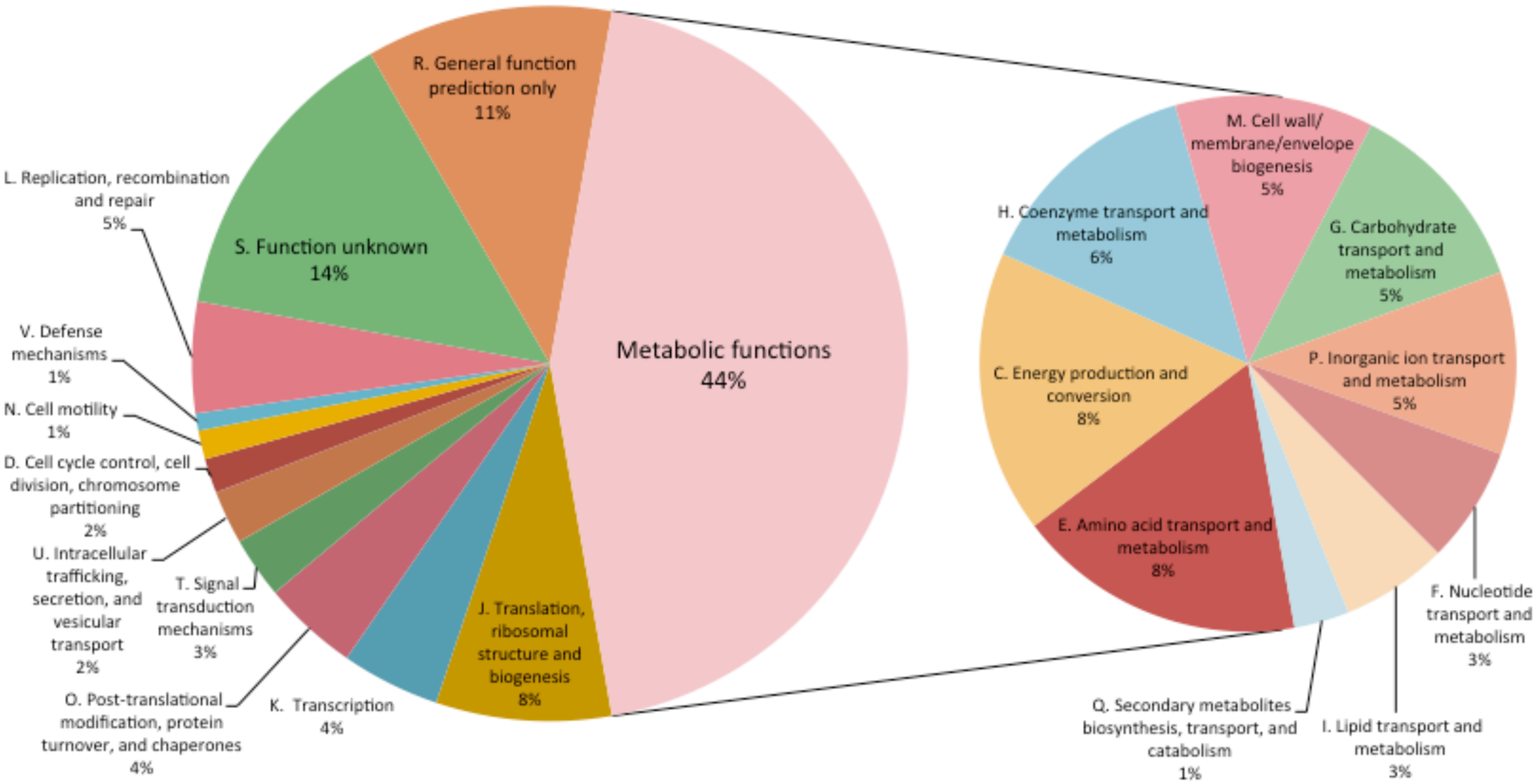

S1 Figure. COG functional categories for prokaryotic essential genes in DEG. COG metabolic functional categories are less detailed than those used in the annotation of metabolic models: both the "Energy production and Conversion" and "Amino acid transport and metabolism" functional categories encompass several of those that are detailed within GSMs, except for the "Transport" category lumped in GSMs, and distributed for each of the major biomolecules in the COG system. Misleading COG annotations included thiO, an essential gene for the biosynthesis of thiamine diphosphate annotated in category E (Amino acid transport and metabolism) and csd, a cysteine desulfurase, essential in 7 datasets and involved in the formation of Fe-S clusters, also annotated in category E.
