# Supplementary material for: Metabolic models and gene essentiality data reveal essential and conserved metabolism in prokaryotes
Source: PLoS Comput Biol. 2018 Nov 16;14(11):e1006556. doi: 10.1371/journal.pcbi.1006556 (PMC6283598; doi:10.1371/journal.pcbi.1006556)
Supplement: S1 Table — The list includes genes conserved in all 79 prokaryotic genomes analysed. Essentiality is given as the number of datasets in DEG (out of 36) in which each gene is essential. The description is that of the corresponding annotated ORF in the genome of E. coli K12. (PDF) [file pcbi.1006556.s003.pdf]

S1 Table. Ubiquitous transporter genes. The list includes genes conserved in all 79 prokaryotic genomes analysed. Essentiality is given as the number of datasets in DEG (out of 36) in which each gene is essential. The description is that of the corresponding annotated ORF in the genome of *E. coli K12*.

Gene name	Description (*E. coli* K12)	Essentiality
alsA	D-allose ABC transporter ATPase	0
araG	L-arabinose ABC transporter ATPase	1
artP	arginine ABC transporter ATPase	1
ccmA	heme export ABC transporter ATPase	2
cydC	glutathione/cysteine ABC transporter export permease/ATPase	7
cydD	glutathione/cysteine ABC transporter export permease/ATPase	3
cysA	Sulfate/thiosulfate import ATP-binding protein	0
ddpD	D,D-dipeptide ABC transporter ATPase	0
ddpF	D,D-dipeptide ABC transporter ATPase	0
dppD	dipeptide/heme ABC transporter ATPase	3
dppF	dipeptide/heme ABC transporter ATPas	1
fecE	Fe(3+) dicitrate transport ATP-binding protein	0
fepC	Ferric enterobactin transport ATP-binding protein	2
fhuC	iron(3+)-hydroxamate import ABC transporter ATPase	0
glnQ	glutamine transporter subunit	0
gltL	glutamate/aspartate ABC transporter ATPase	1
gsiA	glutathione ABC transporter ATPase	0
hisP	histidine ABC transporter ATPase	0
livF	branched-chain amino acid ABC transporter ATPase	0
livG	branched-chain amino acid ABC transporter ATPase	0
malK	maltose ABC transportor ATPase	0
metN	DL-methionine transporter subunit	0
mglA	methyl-galactoside ABC transporter ATPase	1
modC	Molybdenum import ATP-binding protein	1
nikD	Nickel import ATP-binding protein	0
nikE	Nickel import ATP-binding protein	0
potA	spermidine/putrescine ABC transporter ATPase	3
potG	putrescine ABC transporter ATPase	0
proV	glycine betaine/proline ABC transporter periplasmic binding protein	0
pstB	Phosphate import ATP-binding protein	2
rbsA	D-ribose ABC transporter ATPase	0
sapD	Peptide transport system ATP-binding protein	2
ssuB	aliphatic sulfonate ABC transporter ATPase	0
tauB	taurine ABC transporter ATPase	0
thiQ	thiamine/thiamine pyrophosphate ABC transporter ATPase	1
ugpC	sn-glycerol-3-phosphate ABC transporter ATPase	2
xylG	D-xylose ABC transporter dual domain ATPase	0
ydcT	putative ABC transporter ATPase	1
yehX	putative ABC transporter ATPase	0
ytfR	putative sugar ABC transporter ATPase	0
znuC	Zinc import ATP-binding protein	0
