# Supplementary material for: Metabolic models and gene essentiality data reveal essential and conserved metabolism in prokaryotes
Source: PLoS Comput Biol. 2018 Nov 16;14(11):e1006556. doi: 10.1371/journal.pcbi.1006556 (PMC6283598; doi:10.1371/journal.pcbi.1006556)
Supplement: S2 Fig — (PDF) [file pcbi.1006556.s002.pdf]

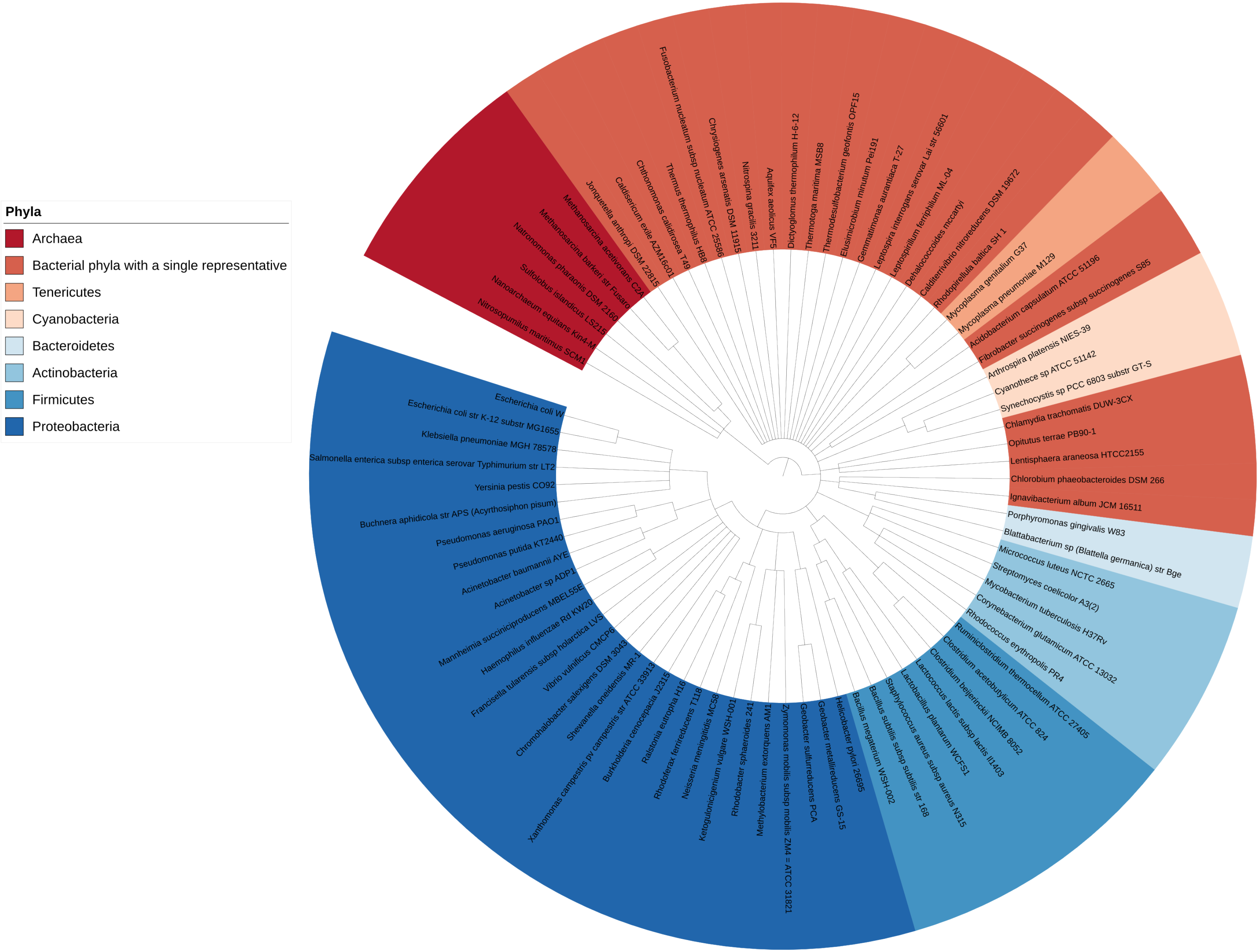

S2 Figure. **Phylogenetic reference tree for species used in the analysis of conservation of essential genes (based on NCBI taxonomy).**
