# Supplementary material for: Metabolic models and gene essentiality data reveal essential and conserved metabolism in prokaryotes
Source: PLoS Comput Biol. 2018 Nov 16;14(11):e1006556. doi: 10.1371/journal.pcbi.1006556 (PMC6283598; doi:10.1371/journal.pcbi.1006556)
Supplement: S2 Table — Essentiality is given as the number of datasets in DEG (out of 36) in which each gene is essential. Conservation is given as the percentage of the 79 genomes where a significant homolog for this gene was found. The description is that of the corresponding annotated ORF in the genome of E. coli K12. Biosynthesized cofactors were manually retrieved from the detailed information available in the Metacyc database. (PDF) [file pcbi.1006556.s004.pdf]

S2 Table – Highly conserved cofactor biosynthesis genes in prokaryotic genomes. Essentiality is given as the number of datasets in DEG (out of 36) in which each gene is essential. Conservation is given as the percentage of the 79 genomes where a significant homolog for this gene was found. The description is that of the corresponding annotated ORF in the genome of E. coli K12. Biosynthesized cofactors were manually retrieved from the detailed information available in the Metacyc database.

Gene Name	Conservation	Description	Cofactor	Essentiality (DEG)
**gltX**	100%	Glutamate-tRNA ligase	Heme	28
**sufC**	100%	Probable ATP-dependent transporter SufC	FeS clusters	2
**sufS**	98.7%	cysteine desulfurase, stimulated by SufE; selenocysteine lyase, PLP-dependent	FeS clusters	2
**iscS**	97.5%	cysteine desulfurase (tRNA sulfurtransferase), PLP-dependent	FeS clusters	10
**entA**	97.5%	2,3-dihydro-2,3-dihydroxybenzoate dehydrogenase	siderophores	0
**ispB**	96.2%	octaprenyl diphosphate synthase	quinones	12
**ispA**	96.2%	geranyltranstransferase	quinones	9
**ispU**	96.2%	undecaprenyl pyrophosphate synthase	quinones	2
**glyA**	94.9%	serine hydroxymethyltransferase	Folates	14
**hemL**	94.9%	glutamate-1-semialdehyde aminotransferase (aminomutase)	porphyrins	14
**bioA**	94.9%	7,8-diaminopelargonic acid synthase, PLP-dependent	Biotin	3
**folD**	94.9%	Bifunctional protein FolD	Folates	19
**dxs**	92.4%	1-deoxyxylulose-5-phosphate synthase, thiamine triphosphate-binding, FAD-requiring	thiamine; isoprenoids;	16
**nadK**	92.4%	NAD kinase	nad/nadp	0
**pdxB**	92.4%	erythronate-4-phosphate dehydrogenase	pyridoxal-5-p	2
**pabA**	92.4%	aminodeoxychorismate synthase, subunit II	Folates	0
**epd**	92.4%	D-erythrose 4-phosphate dehydrogenase	pyridoxal-5-p	1
**gor**	91.1%	glutathione oxidoreductase	glutathione	2
**ribB**	91.1%	3,4-dihydroxy-2-butanone-4-phosphate synthase	riboflavin	5
**ribF**	91.1%	bifunctional riboflavin kinase/FAD synthetase	flavins	15
**ribD**	89.9%	fused diaminohydroxyphosphoribosylamino pyrimidine deaminase and 5-amino-6-(5-phosphoribosylamino) uracil reductase	riboflavin	8
**ribE**	89.9%	riboflavin synthase beta chain	riboflavin	7
**entC**	89.9%	isochorismate synthase 1	terpenoids	1
**ligA**	89.9%	DNA ligase, NAD(+)-dependent	?	23
**folC**	88.6%	bifunctional folylpolyglutamate synthase/ dihydrofolate synthase	Folates	9
**coaD**	88.6%	pantetheine-phosphate adenylyltransferase	CoA	22
**folP**	88.6%	7,8-dihydropteroate synthase	folates	7
**ribC**	88.6%	riboflavin synthase, alpha subunit	riboflavin	3
